# Editorial Notes

**Published:** 1925-01

**Authors:** 


### Editorial Notes.

**Proposed Open-Air Hospital School for Crippled Children.**

In our issue of July last we drew attention to the value of an open-air hospital school as opposed to existing methods in the treatment of the large group of abnormal or diseased conditions in children comprised in the term Orthopædics, and to the highly successful development of these methods at Zuydcoote.

The initiation of a generously conceived hospital school of this description in our midst, the project of the Bristol Crippled Children's Society, is already well launched and supported by many of our representative citizens and colleagues and has our most cordial sympathy.

Several other large towns already possess a hospital school such as Bristol cannot and will not remain long without. It requires no effort of imagination to realise that, for instance, tuberculous disease of the limbs involves prolonged treatment by open-air methods, and that operative methods when needful should be conducted under the favourable environment of the open-air life. Furthermore, a strong case has been made out for placing early rheumatic heart disease in children under similar open-air school hospital conditions, when rest, education and supervised exercises must be carried out for such prolonged periods. To sacrifice the child's chance of education and training by months spent in hospital wards is a woefully costly method that can only be avoided by combining educational facilities with the resources of a special hospital.

The proposal for which support is now invited is on a modest scale, and represents the irreducible minimum of Bristol's requirements; but it will suffice to demonstrate that this open-air hospital school is conceived on sound lines, and an immense advance on anything that Bristol has hitherto been doing for these suffering children.

In and around the site there should be ample room for expansion in connection with this new institution, so that it may be capable of providing for Bristol, Bath and the Counties of Gloucester, Somerset and Wiltshire, since it is impossible with several separate small hospital schools to afford the complete equipment which involves not only the most skilful consulting expert surgical service, but also the resident surgeons, trained nursing staff, X-ray apparatus, and, of course, the resources for operative treatment, remedial exercises, and so forth. Hence the further development of this hospital school is inevitable in due time; for ere long it will have to serve a much wider area than Bristol and its immediate neighbourhood, because the larger the hospital school and the greater the number of children under treatment in one well-organised school the greater the economy.

**Changes in the Editorial Committee.**

The Editor and the *Journal* Committee greatly regret that Dr. James Swain, Emeritus Professor of Surgery, has resigned his position on the Editorial Committee, after serving from 1899 to the end of 1924. During more than a quarter of a century he has not only contributed a large number of valuable original articles on surgical matters, but has earned the grateful thanks of the Society for his devoted service in connection with the editing and production of our *Journal*. We wish him many years of happiness in his new home near-by in Somerset.

Mr. E. W. Hey-Groves, F.R.C.S., Professor of Surgery in the University of Bristol, has joined our Editorial Committee, and his wide experience as Editor of the *Journal of Surgery* will be most helpful in our work.

**A Crematorium for Bristol.**

It is proposed to erect a crematorium in our district. Such facilities should certainly be available in this neighbourhood for those who desire that their remains should be cremated instead of being debarred, as very often is the case, by the hitherto unavoidable heavy expense and trouble entailed by cremation at Woking. Much may be urged for clinging to the beautiful ceremony of interment, but it is unseemly and selfish to obstruct the reasonable desire of those who feel cremation has advantages in both practical and sentimental aspects. Whether the new crematorium be under private or municipal control is outside our concern, but the provision of a well-equipped crematorium is long over-due.

---

